# Potato Resistant Starch Type 1 Promotes Obesity Linked with Modified Gut Microbiota in High-Fat Diet-Fed Mice

**DOI:** 10.3390/molecules29020370

**Published:** 2024-01-11

**Authors:** Weiyue Zhang, Nana Zhang, Xinxin Guo, Bei Fan, Shumei Cheng, Fengzhong Wang

**Affiliations:** 1 College of Food Science and Technology, Hebei Agricultural University, Baoding 071000, China; zhangwy921@163.com; 2 Key Laboratory of Agro-Products Processing, Ministry of Agriculture, Institute of Food Science and Technology, Chinese Academy of Agricultural Sciences, Beijing 100090, China; zhangnn16@163.com (N.Z.); guoxx26@163.com (X.G.); fanbei@caas.cn (B.F.)

**Keywords:** potato resistant starch type 1, obesity, inflammation, gut microbiota, short-chain fatty acids

## Abstract

Obesity has become a major disease that endangers human health. Studies have shown that dietary interventions can reduce the prevalence of obesity and diabetes. Resistant starch (RS) exerts anti-obesity effects, alleviates metabolic syndrome, and maintains intestinal health. However, different RS types have different physical and chemical properties. Current research on RS has focused mainly on RS types 2, 3, and 4, with few studies on RS1. Therefore, this study aimed to investigate the effect of RS1 on obesity and gut microbiota structure in mice. In this study, we investigated the effect of potato RS type 1 (PRS1) on obesity and inflammation. Mouse weights, as well as their food intake, blood glucose, and lipid indexes, were assessed, and inflammatory factors were measured in the blood and tissues of the mice. We also analyzed the expression levels of related genes using PCR, with 16S rRNA sequencing used to study intestinal microbiota changes in the mice. Finally, the level of short-chain fatty acids was determined. The results indicated that PRS1 promoted host obesity and weight gain and increased blood glucose and inflammatory cytokine levels by altering the gut microbiota structure.

## 1. Introduction

Obesity, a primary disease, is a danger to human health globally [1]. According to epidemiological data, obesity has become the fifth leading cause of death worldwide. In 2016, approximately 13% of the global adult population was obese, with a prevalence rate of 11% for males and 15% for females [2]. The International Diabetes Federation reported that there were 451 million adults with diabetes worldwide, with a projected increase to 693 million by 2045 [3]. Obesity and diabetes affect a wide range of people and a large age range, thereby becoming important diseases endangering human health. Interestingly, many studies have shown that dietary interventions can reduce the prevalence of obesity and diabetes [4].

Resistant starch (RS) refers to a special type of starch that cannot be digested in the small intestine. Rather, it ferments with volatile fatty acids in the colon. Research has shown that RS affects the content of certain metabolic products, such as short-chain fatty acids (SCFAs), by altering the structure and abundance of the gut microbiota. RS has beneficial effects on weight loss, thereby lowering blood glucose levels and alleviating inflammation [5,6]. This type of starch may also affect the occurrence or progression of cancer by altering circulating hormone levels and other factors [7]. RS has been shown to affect endogenous intestinal hormone release and improve appetite control and blood glucose control [8].

The differences in RS types arise from differences in food sources, molecular structures, and physicochemical properties. Therefore, their physiological functions also differ. Type 2 RS (RS2) is a dietary fiber composed solely of glucose. Research has shown that RS2 treatment can reverse weight gain, liver steatosis, inflammation, and increased intestinal permeability caused by a high-fat diet (HFD)-influenced changes in the gut microbiota and metabolites [9]. Indeed, RS2 intervention increased the α-diversity of the gut microbiota and promoted *Brucella*, *Bifidobacterium*, and other organisms in the gut microbiota. Meanwhile, RS2 reduced the abundance of *Desulfovibrio*, *Helicobacter*, and *Enterococcus*, which are associated with obesity, inflammation, and aging [10,11]. Research has shown that intervention with RS type 3 (RS3) can reduce host weight and food intake as well as lower blood lipid levels and liver fat accumulation [12]. RS3 has a dose-dependent regulatory effect on HFD-induced obesity-related metabolic syndrome by promoting the proliferation of intestinal cells and expression of tight junction proteins such as occludin and (ZO)-1 [13]. In addition, RS3 promotes the production of microbial metabolites such as propionic acid and acetic acid by regulating the relative abundance of certain gut microbiota, including *Bifidobacterium*, *Ruminococcus*, and *Bacteroidetes* and reducing the abundance of harmful bacteria such as *Escherichia coli* and *Shigella* [14]. Research has shown that RS type 4 (RS4) significantly increases the abundance of *Actinobacteria*, *Bacteroidetes*, and *Bifidobacteria* and reduces the abundance of *Firmicutes* [15]. RS4 intervention reduced the level of cholesterol, fasting blood glucose, and proinflammatory factors in the blood and increased the content of fasting fatty acids such as butyrate, propionate, and valerate in feces [16]. Finally, research has shown that RS type 5 (RS5) has a significant effect in alleviating postprandial hyperglycemia in mice [17]. At the same time, RS5 was shown to alleviate weight gain, reduce the fasting blood glucose level, reduce triglyceride and cholesterol levels in the serum, and increase high-density lipoprotein levels in diabetic mice [18]. These findings suggest that RS has beneficial physiological functions, but significant gaps in research on RS type 1 (RS1) remain.

Because potatoes contain high RS levels, this study examined the effect of potato RS1 (PRS1) on mouse body weights, blood glucose levels, and inflammatory responses. Furthermore, the gut microbiota and metabolites were analyzed to improve our understanding of RS1. This study aimed to provide insights into antagonistic starch and offer a more theoretical basis for dietary intervention.

## 2. Results

### 2.1. Effect of PRS1 on Body Weight, Tissue Weight, and Food Intake

**Figure 1:**
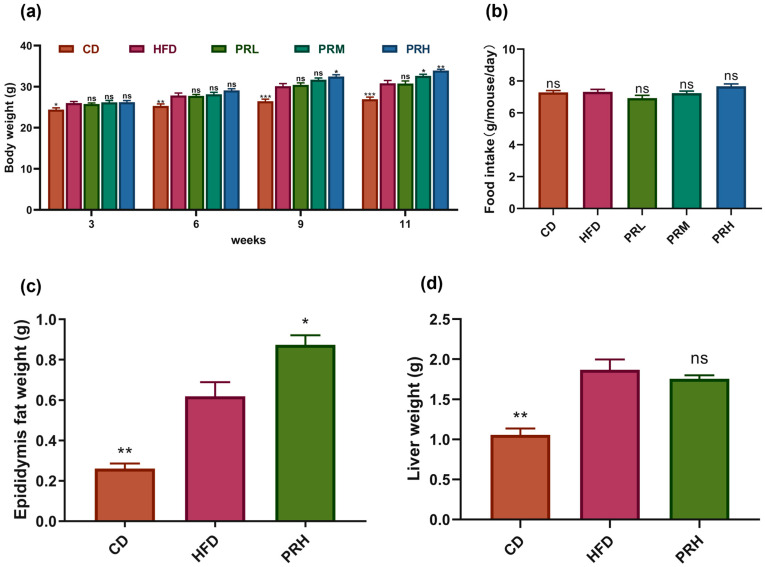
Effect of PRS1 on mouse body weight (**a**), food intake (**b**), epididymal fat weight (**c**), liver weight (**d**). CD: common diet group; HFD: high-fat diet group; PRL: PRS1 low-dose group; PRM: PRS1 medium-dose group; PRH: PRS1 high-dose group. Compared with the HFD group, * *p* < 0.05; ** *p* < 0.01, *** *p* < 0.001; ns *p* > 0.05.

The body weight and fat weight of mice are important indicators for judging obesity. We examined how PRS1 affected diet-induced obesity in C57BL6J mice that were fed a control diet or high-fat diet supplemented with three doses of PRS1 (5, 15, or 25 g/100 g diet) according to previous research [14]. The high-fat diet led to a marked increase in body weight compared to the control diet (Figure 1a). Throughout 11 weeks of treatment, there was no difference in weight gain between PRL receivers and HFD receivers, as shown in Figure 1a. Body weight was slightly, yet not significantly, increased in PRM mice (*p* = 0.1), while the highest dose of PRS1 promoted weight gain significantly (*p* < 0.05) at week 9. At the end of the experiment, PRH receivers still showed a stronger weight gain (*p* = 0.0007) than PRM receivers (*p* = 0.05) in comparison with HFD-fed control mice. These data suggested that PRH-treated mice had a strong tendency for higher body weight gain than that of PRM-treated mice in the experiment. It is reasonable to suppose that the supplementation of 25% PRS1 would result in a strong degree of the indices of metabolic syndrome that would be amenable to the study of the mechanism. As a result, we sought to select the highest dose of PRS1 to study how PRS1 is impacting obesity.

No significant differences were found in the daily food intake for each group (Figure 1b). This result indicated that even with the same level of food intake, the PRS1 intervention groups gained weight more efficiently than the HFD group and that this effect was significant. The most obvious effect occurred in the PRH group. We subsequently chose the PRH group as the treatment group for our research. The results for the epididymal and brown adipose tissue weights showed that the epididymal adipose and liver tissue weights (Figure 1c,d) were significantly increased in the HFD group compared with the CD group (*p* < 0.01). The epididymal fat weights were significantly higher in the PRH group than in the HFD group (*p* < 0.05), and there was an upward trend in the liver weight. These results indicated that PRS1 intervention increased mainly the adipose tissue weight and that its effect on the liver was not obvious.

### 2.2. Effect of PRS1 on Glucose Metabolism in HFD-Fed Mice

**Figure 2:**
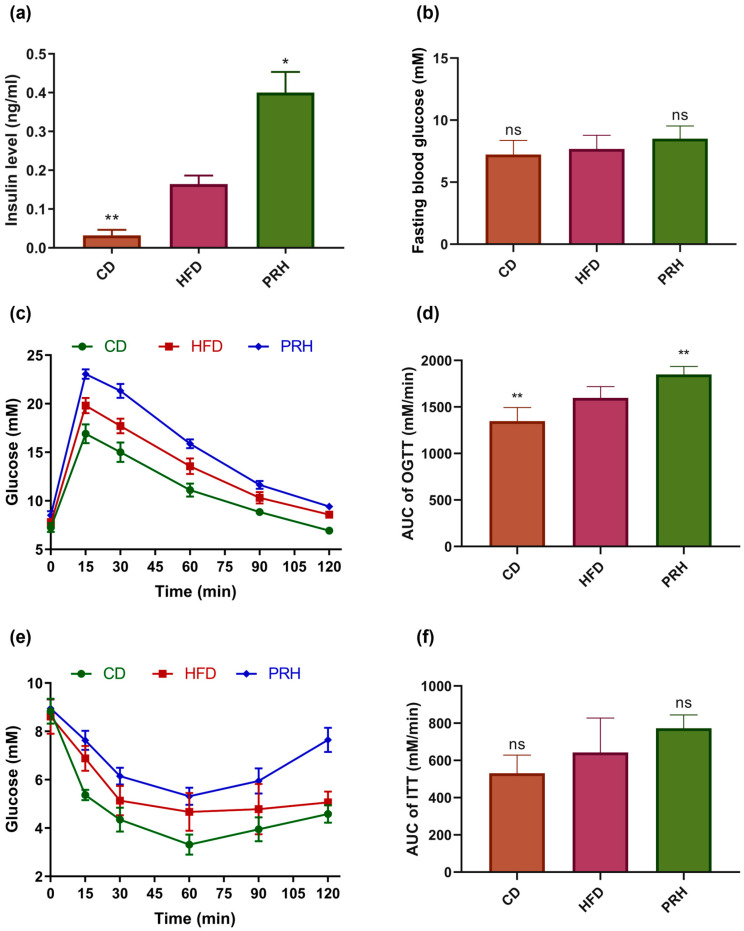
Effect of PRS1 on mouse blood insulin (**a**), fasting blood glucose (**b**), OGTT (**c**), AUC of OGTT (**d**), ITT (**e**), and AUC of ITT (**f**). CD: common diet group; HFD: high-fat diet group; PRH: PRS1 high-dose group. Compared with the HFD group, * *p* < 0.05; ** *p* < 0.01; ns *p* > 0.05.

The insulin content in the blood of mice was detected, and the oral glucose tolerance test (OGTT) and insulin tolerance test (ITT) were evaluated to determine the effect of HFD on blood glucose levels and insulin secretion in mice. Our results indicated that the HFD group had significantly increased blood insulin levels. Compared with the HFD group, the PRH group had a more significant increase in blood insulin levels (Figure 2a). OGTT was performed with the assumption that there were no significant differences in fasting blood glucose levels between the groups (Figure 2b). The OGTT results showed that in each group, the peak blood glucose level was reached at the 15th min after glucose stimulation. Compared with the HFD group, the PRH group had a more significant glucose increase (Figure 2c). After the 15th min, the blood glucose level of all three groups decreased. Compared to the CD group, both the HFD and PRH groups had higher area under the curve (AUC) values (Figure 2d). Furthermore, the AUC was significantly higher in the PRH group than in the HFD group (*p* < 0.01). The insulin tolerance test (ITT) results showed that at 60 min after insulin injection, the blood glucose level of each group gradually increased (Figure 2e). In particular, the PRH group had a higher AUC than the HFD group (Figure 2f). These results indicated that PRS1 intervention weakened the ability of the mice to control their blood glucose levels, which led to insulin resistance.

### 2.3. Effect of PRS1 on Lipid Profile and Serum Inflammatory Factors

**Figure 3:**
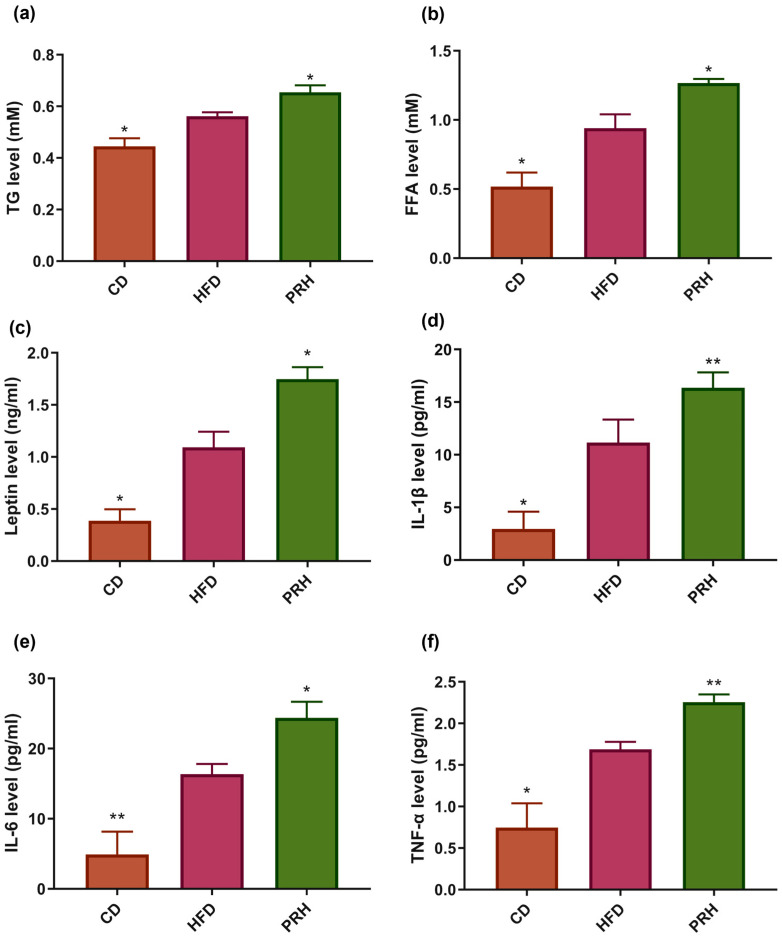
Effect of PRS1 on mouse serum level of TG (**a**), FFA (**b**), leptin (**c**), IL-1β (**d**), IL-6 (**e**), and TNF-α (**f**). CD: common diet group; HFD: high-fat diet group; PRH: PRS1 high-dose group. Compared with the HFD group, * *p* < 0.05; ** *p* < 0.01.

The effect of PRS1 on obesity in mice was evaluated by detecting the level of blood glucose, lipid-related indicators, and inflammatory factors in the blood. The results showed that serum total glyceride (TG) and free fatty acid (FFA) levels were significantly higher in the PRH group than in the HFD group (Figure 3a,b). It is known that adipose tissue growth in mice can cause an increase in serum leptin levels [19,20]. In this study, leptin levels in the blood also showed a significant upward trend (Figure 3c). Compared with the HFD group, the PRH group had a significantly increased serum level of IL-6 (Figure 3e) and significantly increased serum levels of IL-1β and TNF-α (Figure 3d,f). These results indicated that PRS1 not only increased blood TG and FFA levels in mice but also caused an inflammatory response and leptin resistance.

### 2.4. Effect of PRS1 on the Micromorphology of White Adipose, Brown Adipose, and Liver Tissues

**Figure 4:**
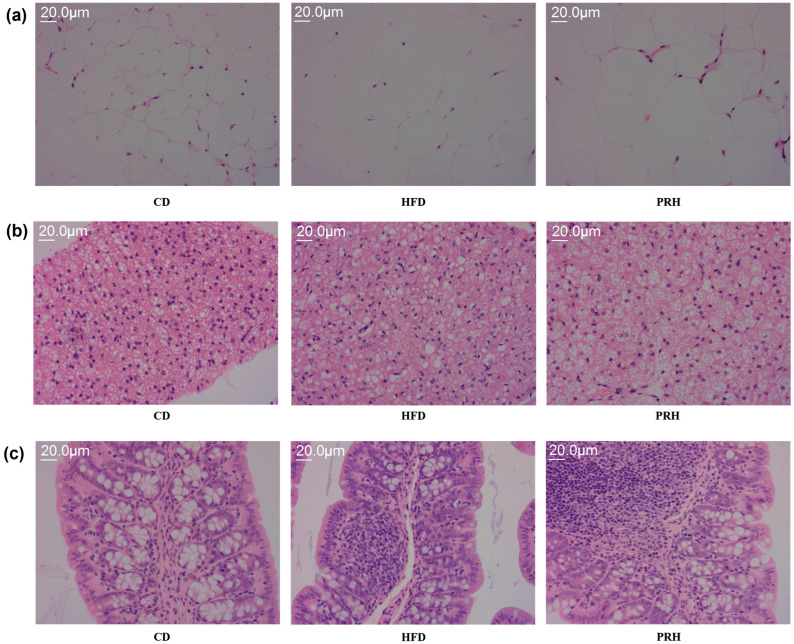
H&E staining of white adipose tissue (**a**), brown adipose tissue (**b**), and colon tissue (**c**). CD: common diet group; HFD: high-fat diet group; PRH: PRS1 high-dose group.

H&E staining was performed to visually assess the effect of PRS1 intervention on mouse adipose tissue, liver tissue, and colon tissue. Compared with the model group, the HFD group exhibited an increased average size of white adipocytes and a reduced number of brown adipocytes (Figure 4a,b). PRS1 intervention alleviated the HFD-induced damage to the mouse colon tissue structure (Figure 4c). These findings indicated that PRS1 intervention accelerated the growth of white adipose tissue, weakened the thermogenic capacity of the original brown adipose tissue, and probably had adverse effects on liver health.

### 2.5. PRS1 Increases Inflammatory Factor Expression and Intestinal Permeability in Colon Tissue

**Figure 5:**
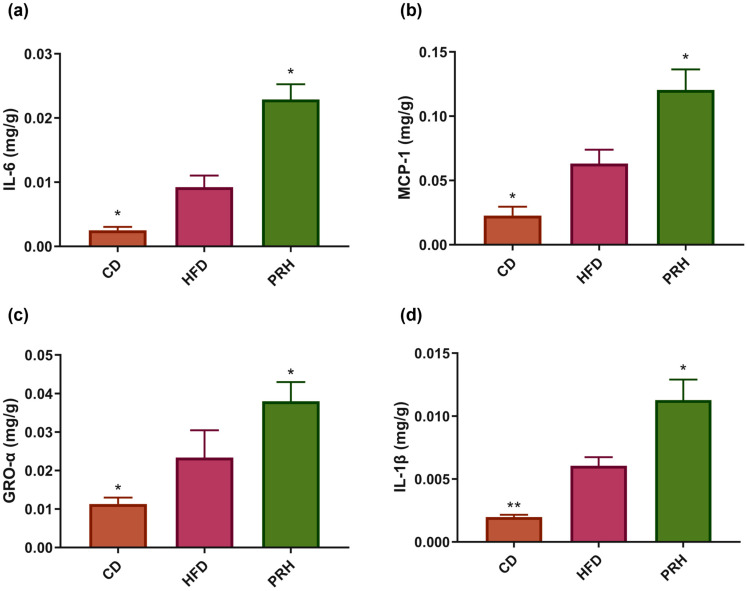

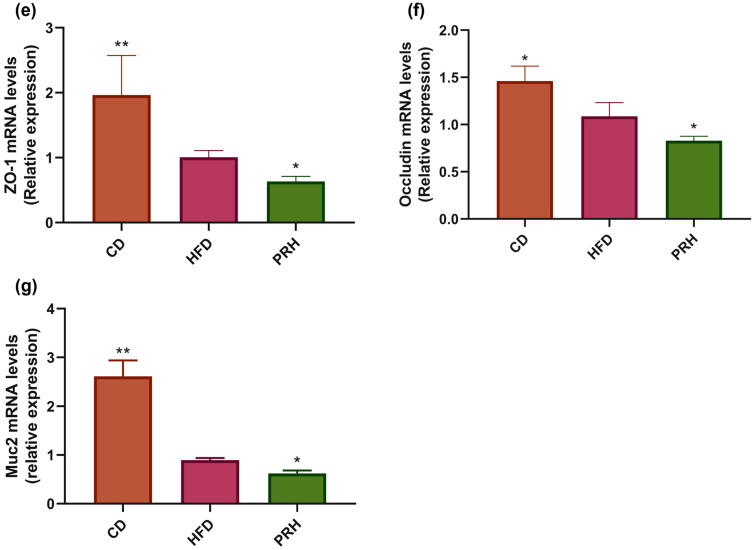
Level of IL-6 (**a**), MCP-1 (**b**), GRO-α (**c**), IL-1β (**d**), ZO-1 relative expression (**e**), occludin relative expression (**f**), and Muc2 relative expression (**g**). CD: common diet group; HFD: high-fat diet group; PRH: PRS1 high-dose group. Compared with the HFD group, * *p* < 0.05; ** *p* < 0.01.

Inflammatory factors in the colon tissue were investigated to explore the effect of PRS1 on colonic inflammatory response. The results of inflammatory factor levels in the colon and the expression of genes related to intestinal permeability are shown in Figure 5. Compared with the CD group, the HFD group had lower levels of IL-6, MCP-1, GRO-α, and IL-1β. The inflammatory factor levels in the PRH group showed an upward trend, and there were significant differences compared to the HFD group (Figure 5a–d). The gene expression level of occludin, which is related to intestinal permeability, was significantly decreased in the PRH group compared to the HFD group (*p* < 0.05) (Figure 5e–g). Additionally, ZO-1 and Muc2 gene expression levels were significantly decreased (*p* < 0.01). The above results indicated that PRS1 intervention did not alleviate the inflammatory response induced by HFD. In contrast, the intervention damaged the intestinal colon barrier and increased its permeability.

### 2.6. PRS1 Changes the Abundance and Diversity of the Intestinal Flora in Mice

RS is fermented and broken down by the colonic bacterial community. Therefore, the gut microbiota in the colon was analyzed to explore its effect on obesity in mice.

#### 2.6.1. Alpha Diversity Analysis

**Figure 6:**
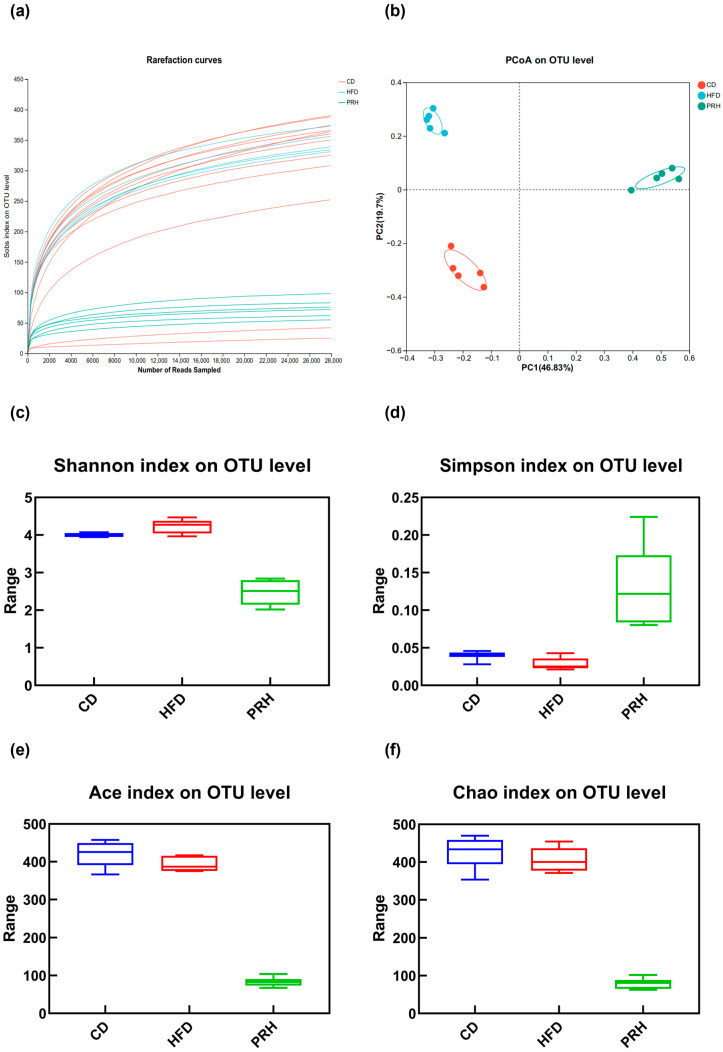

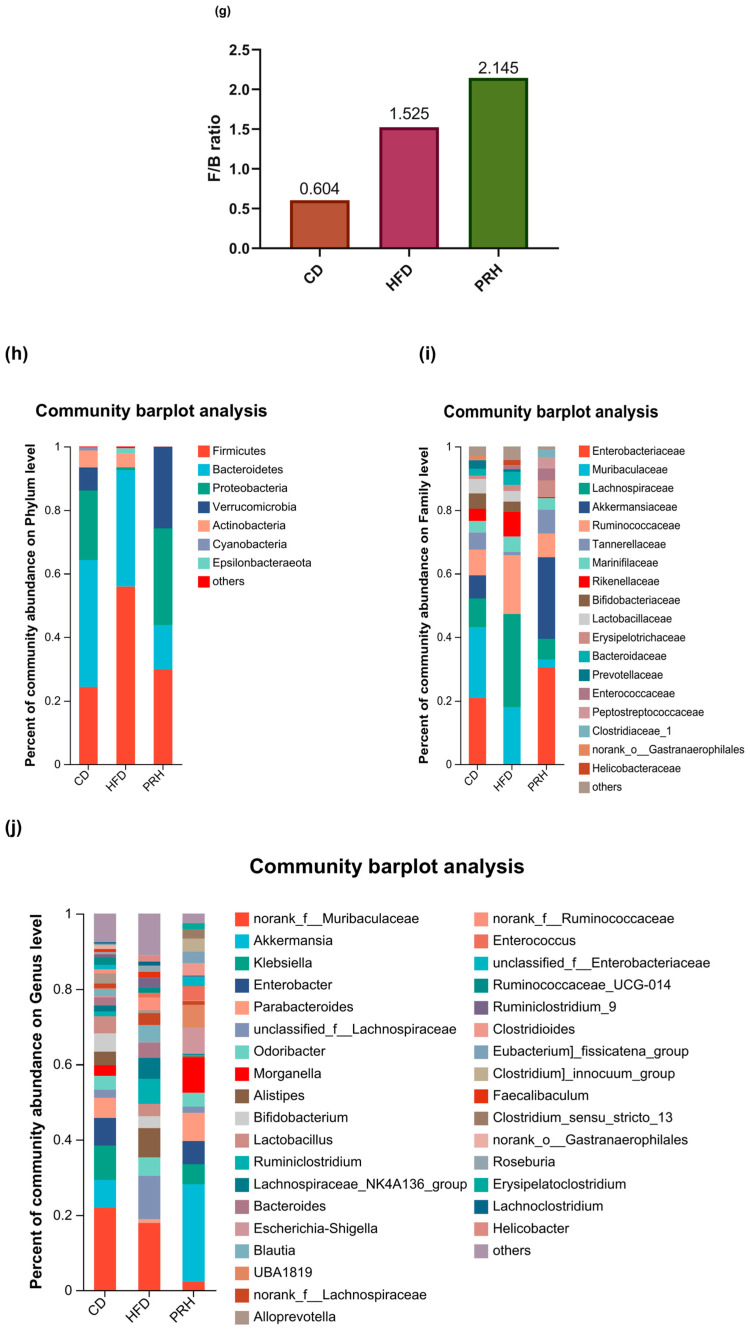
Rarefaction curve (**a**), PCoA (**b**), Shannon index (**c**), Simpson index (**d**), Ace index (**e**), Chao index (**f**), Firmicutes/*Bacteroidetes* (F/B) ratio (**g**), phylum level species composition (**h**), family level species composition (**i**), and genus level species composition (**j**). CD: common diet group; HFD: high-fat diet group; PRH: PRS1 high-dose group.

Rarefaction curves can directly reflect the rationality of sequencing data and indirectly reflect the richness of species in a sample [21]. Our results (Figure 6a) showed that the species diversity was far lower in the PRH group than in the CD and HFD groups. In addition, the dilution curves of the three groups indicated that Group C tended to be flat at 22,000 OTUs, Group M tended to be flat at 14,000 OTUs, and Group PRH tended to be flat at 12,000 OTUs. These findings indicated that the sequencing data volume was reasonable and that a greater volume of data can only produce a small number of new OTUs.

Alpha diversity is an ecological indicator that shows how many taxonomic groups exist in each sample and whether the abundance of these groups is evenly distributed [22]. The dietary interventions of functional foods that increase alpha diversity and change the abundance of specific bacteria can significantly change the fecal microbiota, independent of antidiabetes drugs [23]. We used Mothur’s method to calculate and evaluate the gut microbiota abundance after PRS1 dietary intervention. The alpha diversity and community diversity indexes were analyzed by principal coordinate analysis (PCoA) using the Bray–Curtis distance, and the microbial community composition of each group before and after the intervention was compared at the phylum, family, and genus levels [24].

This experiment analyzed the abundance (Ace and Chao indexes) and diversity of the gut microbiota (Shannon and Simpson indexes). The Shannon index was higher for the PRH group than the HFD group, but the Simpson index was lower for the PRH group than the HFD group (Figure 6c,d). These data indicated that PRS1 intervention improved the richness of the gut microbiota community in the mice but reduced the uniformity of the community. At the same time, the Ace and Chao indexes were significantly lower for the PRH group than the HFD group (Figure 6e,f). We thus concluded that PRS1 intervention reduced the evenness of the intestinal microbial community of mice and ultimately reduced the species diversity of the flora.

#### 2.6.2. Beta Diversity Analysis

A PCoA was conducted for the gut microbiota to examine the mechanism of microbial community composition changes between the different groups. The results showed that compared with no intervention in the HFD group, increased PRS1 administration resulted in significant changes in the intestinal microbiota composition (Figure 6b). The changes observed for PC1 and PC2 were 46.83% and 19.7%, respectively.

#### 2.6.3. Gut Microbiota Composition

Sample level clustering analyses at the phylum, family, and genus classification levels revealed the following (all compared to the HFD group). At the phylum level, PRS1 intervention increased the ratio of *Firmicutes* to *Bacteroidetes* (F/B ratio) and the abundance of *Verrucomimicrobia* (Figure 6g,h). At the family level, the abundance of *Muribacillaceae*, *Lachnospiraceae*, *Tannellaceae*, *Marinifilaceae*, *Rikenellaceae*, *Lactobacillaceae*, and *Bacteroidaceae* significantly decreased in the PRH group and that of *Akkermansiaceae*, *Enterobacteriaceae*, *Erysipelotrichaceae*, *Peptostreptococcaceae*, and *Clostridiaceae_1* significantly increased (Figure 6h). At the genus level, the abundance of *norank_f_Muribaculaceae*, *unclassified_f_Lachnospiraceae*, *Odoribacter*, *Bifidobacterium*, *Alistipes*, *Ruminiclostridium*, *Lactobacillus*, and *Bacteroides* decreased in the PRH group, while the abundance of *Akkermansia*, *Morganella*, *Escherichia-Shigella*, *Enterobacter*, *UBA1819*, *Klebsiella*, and *Clostridioides* increased (Figure 6i).

### 2.7. PRS1 Reduces SCFA Secretion

**Figure 7:**
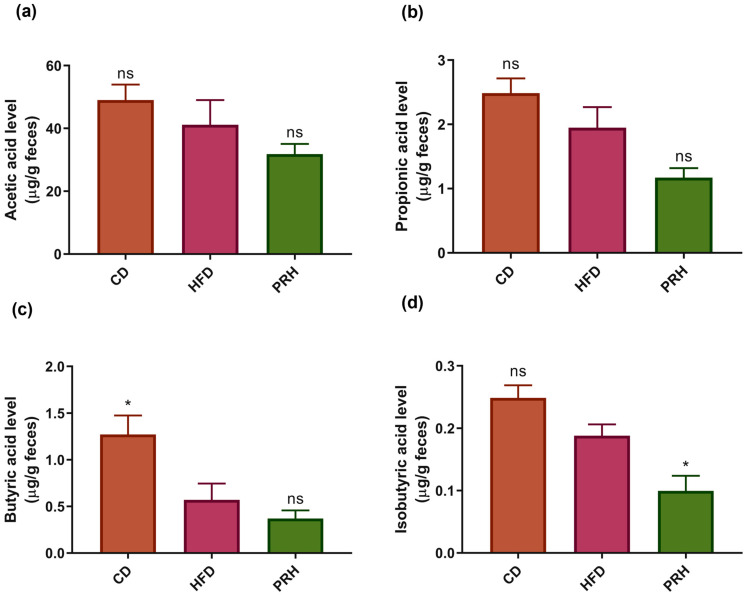
Level of acetic acid (**a**), propionic acid (**b**), butyric acid (**c**), and isobutyric acid (**d**). CD: common diet group; HFD: high-fat diet group; PRH: PRS1 high-dose group. Compared with the HFD group, * *p* < 0.05; ns *p* > 0.05.

The fermentation and decomposition of PRS1 by gut microbiota can produce various metabolites. Previous studies have shown that SCFAs are closely related to obesity in mice [25,26]. According to previous research, RS enters the intestine and is fermented by microorganisms, thereby producing a series of metabolic products [27,28]. Our SCFA results are shown in Figure 7. The level of acetic acid, propionic acid, butyric acid, and isobutyric acid followed a decreasing trend in the HFD group compared with the CD group. Compared with the HFD group, the PRH group had lower levels of SCFAs. This result indicated that PRS1 intervention reduced SCFA secretion, promoted weight gain, and caused inflammation in mice.

## 3. Discussion

### 3.1. Effect of PRS1 on HFD-Induced Obesity and Inflammatory Responses

This study showed that PRS1 intervention led to increases in body and fat weights. The average weekly food intake of each mouse was nearly the same, and these results indicated that PRS1 increased the body and fat tissue weights of mice consuming HFD. Additionally, this intervention had a dose-dependent effect. H&E staining results showed that PRS1 accelerated the accumulation of white adipose tissue and reduced the amount of brown adipose tissue. PRS1 weakened the fat consumption and heat production of the mice and led to weight gain and obesity. However, PRS1 may have adverse effects on liver health. The OGTT and ITT results showed that PRS1 reduced the ability of the mice to control their blood glucose levels, as evidenced by increased blood glucose levels and insulin resistance.

### 3.2. Effect of PRS1 on Blood Lipid Levels, Inflammatory Response, and Intestinal Barrier in HFD Mice

The PRS1 intervention increased the serum TG and FFA levels in the mice. Leptin, which is positively correlated with fat content, was also significantly increased. The serum levels of the inflammatory factors IL-1β, TNF-α, and IL-6 increased as well as the levels of the inflammatory cytokines IL-6, MCP-1, GRO-α, and IL-1β in the colon. PCR amplification analysis showed that the expressions of ZO-1, occludin, and Muc2, which are related to epithelial cell junction and intestinal barrier integrity, all decreased. These results indicated that inflammation occurred in the colon. PRS1 disrupted the integrity of the colon epithelium, causing damage to the intestinal barrier.

### 3.3. Effect of PRS1 on the Gut Microbiota Composition of HFD-Fed Mice

According to previous research findings, disrupted glucose and lipid metabolism and inflammatory responses in mice are closely related to the intestinal microbiota composition. This study investigated gut microbiota changes at three taxonomic levels: phylum, family, and genus. At the phylum level, previous studies have shown that dietary fiber and probiotics can increase the relative abundance of *Actinobacteria* and *Bacteroidetes* and reduce the relative abundance of Firmicutes, thereby preventing obesity. However, the results of this study showed a decreased abundance of *Firmicutes*, *Bacteroidota*, and *Actinobacteria*, indicating that PRS1 did not play a role in preventing obesity.

The *Firmicutes*/*Bacteroidetes* (F/B) ratio is considered a biomarker of weight loss [29,30,31], and the human gut microbiota is composed mainly of these two dominant phyla, which account for over 90% of the total microbiota, as well as some other subdominant phyla [32]. Many studies have shown that the F/B proportion was higher in obese individuals than in lean individuals and that this ratio decreased with weight loss. Further studies have shown a positive correlation between the fecal concentration of Bacteroides and body mass index [33], indicating that high F/B ratio values are often associated with obesity. In this study, the F/B ratio was significantly higher in the PRH group than in the HFD group, and the overall abundance was decreased. In addition, the abundance of Proteobacteria, a biomarker of ecological imbalance and risk, also followed an increasing trend [34]. These data indicated that PRS1 not only had no preventive effect on obesity but also caused a certain degree of damage to the original microbial community, thereby increasing the risk of obesity in HFD-fed mice.

At the genus level, studies have shown a negative correlation between intestinal inflammation and Lactobacillus and a positive correlation with *Ruminococcus* [35,36]. *Ruminococcus* helps alleviate diarrhea and reduces the risk of diabetes and colon cancer [37,38,39]. Studies have shown that *Akkermansia* can control HFD-induced inflammation and body weight in mice and that adding RS to the diet can increase the relative abundance of *Akkermansia* [9,40]. *Bifidobacterium* is an important bacterial genus closely related to human health [41,42]. It can break down RS and accelerate growth, which is negatively correlated with obesity, glucose intolerance, and SCFA production [43].

Research has shown that RS can increase the relative abundance of Bacteroides, which can reduce body weight by fermenting RS and promoting SCFA production, especially acetic acid, in the intestine [44]. Our study indicated that PRS1 intervention increased *Akkermansia* abundance, which is consistent with the previous results documented in the literature. However, the abundance of *Bifidobacterium* decreased, indicating that the PRS1-consuming mice exhibited the symptoms of glucose intolerance and had an increased risk of obesity. By contrast, consuming PRS1 decreased the abundance of Bacteroides, indicating that PRS1 could not indirectly reduce weight or promote intestinal SCFA production through Bacteroides.

### 3.4. Effect of PRS1 Intervention on SCFA Content

According to previous research, RS enters the intestine and is fermented by microorganisms, producing a series of metabolic products. The level of certain SCFAs, such as acetic acid, propionic acid, and butyric acid, is negatively correlated with obesity-related parameters. SCFAs promote physiological benefits in the intestinal epithelium of the host by increasing the weight of the large intestine and cecal tissue. In this study, the levels of acetic acid, propionic acid, butyric acid, and isobutyric acid were decreased. Based on these metabolite results, we assert that PRS1 intervention did not promote weight loss in HFD mice and that its effect on alleviating obesity was not significant.

## 4. Materials and Methods

### 4.1. Mouse Experimental Environment and Groups

PRS1 with a purity of >90% was purchased from Louis Francois Co. (Paris, France). Animal experiments were approved by the Basic Medical Animal Care and Use Committee of Inner Mongolia Medical University (Inner Mongolia, China). The ethical review batch number is “YKD202101103”. For this study, 60 C57BL/6 male mice 5–6 weeks of age were purchased from Vital River Laboratory Animal Technology (Beijing, China). All mice were fed in the animal room, which was maintained at 25 °C ± 1 °C and 55% ± 5% humidity. The animals were exposed to light for 12 h and darkness for 12 h in a day [14]. Prior to the start of the experiment, all mice were allowed to acclimate for 1 week with access to normal food and water. One week later, the mice were divided into six groups, with 10 mice in each group, and each group was placed in a separate cage [45]. The mice were fed with control diet, high-fat diet (HFD), and HFD diet supplemented with 5%, 15%, or 25% PRS1/kg of food mass for 11 weeks. The experimental groups were as follows: CD group (ccontrol diet; 19.2% protein, 4.3% fat, 67.3% carbohydrate, total energy 4057 kcal/kg), HFD group (high-fat diet group; 23.7% protein, 23.6% fat, 41.4% carbohydrate, total energy 4057 kcal/kg), PRL group (PRS1 low-dose group; HFD with 5% PRS1; 23.9% protein, 23.8% fat, 41.8% carbohydrate, total energy 4057 kcal/kg;), PRM group (PRS1 medium-dose group; HFD with 15% PRS1; 22.6% protein, 22.5% fat, 39.6% carbohydrate, total energy 4057 kcal/kg), and PRH (PRS1 high-dose group; HFD with 25% PRS1; 18.8% protein, 18.8% fat, 33.0% carbohydrate, total energy 4057 kcal/kg). The diet composition of all groups used in this study is shown in Table 1. In this study, we first studied the effect of PRS1 on diet-induced weight gain with doses of 5%, 15%, and 25% according to previous research [14]. The supplementation dose of PRS1 that would result in the strongest degree of weight gain was then used to investigate the effect on other indices of metabolic syndrome and the underlying mechanism. The mouse weights and dietary intakes were measured each week.

### 4.2. Mouse Adipose, Colon, and Liver Tissue Samples

The mice were euthanized via the cervical dislocation method. Epididymal adipose, brown adipose, colon, and liver tissues were removed from the mice and frozen in liquid nitrogen. To collect and process colon tissues, 2 mm colon segments were cleaned of their intestinal contents with PBS. Next, the samples were homogenized using a grinder, and after centrifuging the sample at 1500× *g* and 4 °C for 15 min, the supernatant was collected. ELISA kits (Mercodia, Uppsala, Sweden) were used to measure the content of the inflammatory factors in the colon tissues.

### 4.3. Blood Biochemical Indexes

Retro-orbital blood samples were collected from the mice and centrifuged at 1800× *g* for 20 min. The upper serum portions were removed and stored at −80 °C. Total triglyceride (TG), free fatty acid (FFA), insulin, and leptin levels in the blood were detected using ELISA kits (Mercodia, Uppsala, Sweden) according to the manufacturer’s instructions.

### 4.4. OGTT and ITT

Before experiments, the mice were placed in clean cages and fasted with free access to only water for 16 h. Then, the mice were weighed and gavaged with 1 g/kg glucose. Blood was collected from the tail vein at 0, 15, 30, 60, 90, and 120 min after the glucose was administered. The blood glucose concentrations were measured using a glucometer (Roche, Basel, Switzerland) to determine the OGT.

Prior to the next experiment, the mice were fasted for 6 h with free access to only water. Then, the mice were injected intraperitoneally with 0.5 U/kg insulin (Sigma-Aldrich, St. Louis, MO, USA). At 0, 15, 30, 60, 90, and 120 min after insulin administration, tail vein blood samples were collected, and the glucose concentrations were measured as described above to determine the IT.

### 4.5. Hematoxylin and Eosin Staining (H&E)

Mouse tissues were fixed in 4% paraformaldehyde at 4 °C for 24 h, embedded in paraffin, and cut into 4 mm sections. The paraffin sections were stained with hematoxylin for 5 min, rinsed with water, and placed in 1% acetic acid 5 times for 30 s each time. The samples were then rinsed with water for 5 min and stained with eosin [46], followed by rinsing with water for 5 min. A microscope (Olympus, Tokyo, Japan) was used to observe the staining results.

### 4.6. 16S rRNA Gene Sequence Analysis

On the fourth week of the experiment, the mice were placed in metabolic cages, and feces were collected for 24 h. A total of 300–400 mg of feces was collected from each group. The samples were placed in 1-mL sterile centrifuge tubes and quickly transferred to a −80 °C ultra-low temperature freezer for storage. Total microbiome DNA was extracted from the feces using the QIAamp DNA Stool Mini Kit (Qiagen, Hilden, Germany), and the extracted samples were stored at −20 °C. The DNA concentration and purity were assessed using the A260 nm/A280 nm ratio. The ABI GeneAmp^®^ 9700 PCR System (Applied Biosystems, Foster City, CA, USA) was used for PCR amplification [47], and the QuantiFluorTM-ST Handheld Fluorometer with UV/Blue Channels (Promega Corporation, Madison, WI, USA) was used to quantify the amplification product. The collected samples were quantified and sequenced using the Major Bio Pharm Technology Co., Ltd. platform (Shanghai, China). Universal primers 27F (5′-AGAGTTTGATCCTGGCTCAG-3′) and 533R (5′-TTACCGCGGCTGCTGGCAC-3′) were used to amplify the hypervariable V3–V4 regions via the polymerase chain reaction (PCR) of the 16S rDNA gene. The gene sequence was filtered, spliced, and classified into operational taxon units (OTUs) to ensure that the sequencing error rate was <1% and the percentage of bases < 0.01 was >97% [48].

### 4.7. Real Time-qPCR

Colon tissue samples (1 cm in length) were rinsed with PBS buffer (Invitrogen, Carlsbad, CA, USA) to remove the colon contents. After lysis buffer (Solarbio, Beijing, China) was added, the samples were homogenized with a grinder (Servicebio, Wuhan, China). The homogenates were centrifuged at 1500× *g* and 4 °C for 15 min; the supernatants were then collected, and RNA was extracted using a kit (Aidlab, Beijing, China). The DNA concentration and purity were assessed using the A260 nm/A280 nm ratio. Reverse transcription was performed according to the manufacturer’s instructions. cDNA was obtained using a transcription Kit (Aidlab, Beijing, China), and the ABI GeneAmp^®^ 9700 PCR System (Applied Biosystems, Foster City, CA, USA) was used for gene expression detection [49,50,51]. Table 2 lists the primer sequences used in this study.

### 4.8. Statistical Analysis

SPSS statistics 23 (IBM, Armonk, NY, USA) and GraphPad Prism 8.0 (GraphPad Software Inc., San Diego, CA, USA) were used to conduct one-way analysis of variance (ANOVA) and Duncan multiple comparison analysis. The threshold value of significance was set as *p* < 0.05, and *p* < 0.01 was considered extremely significant. Excel 2016 (Microsoft, Washington, DC, USA) was used to calculate the average values of the data, which are expressed as the mean ± standard deviation.

## 5. Conclusions

PRS1 intervention can lead to obesity and weight gain in mice. PRS1 increased blood glucose and lipid levels and led to both insulin and leptin resistance. Furthermore, PRS1 increased serum inflammatory factor levels in mice. It also increased the permeability of the colon, destroyed the intestinal barrier, and increased the level of inflammatory factors in the colon. PRS1 promoted white fat accumulation, reduced the production of brown adipose tissue, and inhibited the transformation and consumption of fat. The abundance of Bacteroides as well as level of SCFA decreased after PRS1 intervention. This suggests that PRS1 does not reduce body weight indirectly or promote SCFA production in the gut through Bacteroides. In this study, physiological indexes, gene expression, intestinal microbiota, and metabolites were analyzed. The results indicated that PRS1 indirectly affected SCFA production by altering the structure of the intestinal flora, thereby promoting host obesity and weight gain.

This study evaluated the effect of RS1 on HFD-induced obesity in mice to provide a more theoretical basis for alleviating obesity and dietary interventions. Differences in the molecular structure and physicochemical properties of different RS types, and their physiological functions are also different. It is needed to address the physiological functional differences between different types of RS in the future.

## Figures and Tables

**Table 1:** The composition of the diet used in this study.

	CD		HFD		PRL		PRM		PRH	
Ingredient	gm	kcal	gm	kcal	gm	kcal	gm	kcal	gm	kcal
Casein	200	800	200	800	200	800	200	800	200	800
L-Cystine	3	12	3	12	3	12	3	12	3	12
Maltodextrin 10	150	600	100	400	100	400	100	400	100	400
Sucrose	0	0	172.8	691.2	172.8	691.2	172.8	691.2	172.8	691.2
PRS1	0	0	0	0	42.5	0	90	0	269	0
Soybean Oil	25	225	25	225	25	225	25	225	25	225
Lard	20	180	177.5	1597.5	177.5	1597.5	177.5	1597.5	177.5	1597.5
Mineral Mix S10026	10	0	10	0	10	0	10	0	10	0
Dicalcium Phosphate	13	0	13	0	13	0	13	0	13	0
Calcium Carbonate	5.5	0	5.5	0	5.5	0	5.5	0	5.5	0
Potassium Citrate, 1 H_2_O	16.5	0	16.5	0	16.5	0	16.5	0	16.5	0
Vitamin Mix V10001	10	40	10	40	10	40	10	40	10	40
Choline Bitartrate	2	0	2	0	2	0	2	0	2	0
Total	1055.1	4057	858.15	4057	850.65	4057	898.15	4057	1077.1	4057

PRS1, potato-resistant starch type 1.

**Table 2:** Reaction primers.

Gene	Forward and Reverse Primers
*ZO-1*	F: 5′-TTTGAGACGACTCGGGGGAT-3′
	R: 5′-TCTCGTTTTCTGGTTGGCAGT-3′
*Occludin*	F: 5′-CGCGTGCACACACACAATAA-3′
	R: 5′-TAGTAACGGAAAGGACCCCC-3′
*Muc2*	F: 5′-GTTTGGACACGCACAAGGAC-3′
	R: 5′-CTCGGGTAGCTTCCACTGTT-3′

## Data Availability

Data are contained within this article.
